# Protective Effects of Resveratrol in Experimental Retinal Detachment

**DOI:** 10.1371/journal.pone.0075735

**Published:** 2013-09-11

**Authors:** Wei Huang, Guorong Li, Jianming Qiu, Pedro Gonzalez, Pratap Challa

**Affiliations:** Duke Eye Center, Duke University, Durham, North Carolina, United States of America; University of Florida, United States of America

## Abstract

**Background:**

Oxidative stress is one of the major factors that trigger photoreceptor apoptosis. To investigate whether resveratrol, a potent antioxidant and small molecule activator of the FoxO pathway, would be neuroprotective against photoreceptor cell death in a rodent model of retinal detachment.

**Methods:**

Retinal detachment was created in adult Brown Norway rats by subretinal injection of sodium hyaluronate. The animals were treated daily with vehicle or resveratrol (20mg/kg) intraperitoneal injection. Photoreceptor death was assessed by counting the number of apoptotic cells with TdT-dUTP terminal nick-end labeling (TUNEL) and measurement of the outer nuclear layer (ONL) thickness 3 days after RD. Changes in expression of FoxO1a, FoxO3a, and FoxO4 were analyzed by western blot. The activity of caspase 3, caspase 8, caspase 9, spectrin and their cleavage forms were studied.

**Results:**

Three days after retinal detachment, caspase 3, caspase 8 and caspase 9 were significantly activated in the detached retina. Spectrin cleavage products at 120 and 145 kDa were also detected. Both caspase and calpain activation are involved in apoptotic photoreceptor cell death in detached retinas. Treatment with resveratrol increases FoxO1a, FoxO3a, and FoxO4 protein expression in detached retinas only. Resveratrol treatment decreases activation of intrinsic and extrinsic caspase apoptotic pathways triggered by RD. The number of TUNEL-positive cells decreases from 1301±51 cells/mm^2^ in control groups to 430±35 cells/mm^2^ in treatment groups (p<0.05). Resveratrol treatment also demonstrates 59% less ONL thickness loss compared to controls.

**Conclusions:**

Resveratrol treatment up-regulates the FoxO family and blocks Caspase3, 8, and 9 activation. Resveratrol has the potential to be used as a novel therapeutic agent for preventing vision loss in diseases characterized by photoreceptor detachment.

## Introduction

Photoreceptors are the primary transducers of visual stimuli and receive the majority of their nutritional and metabolic support from the underlying retinal pigment epithelium (RPE). In a variety of diseases such as retinal detachment (RD), diabetic retinopathy and age-related macular degeneration, neurosensory retina separates from the underlying RPE. Although surgery can be done to reattach the retina, only two fifths of patients with a rhegmatogenous retinal detachment involving the macula recover 20/40 or better vision. Those that lose vision appear to do so because of photoreceptor death [1]. Thus, identification of the mechanisms that underlie photoreceptor death is critical to developing new treatment strategies for these diseases.

Physical separation of photoreceptors from the RPE reduces oxygen and nutrient supply to the photoreceptor outer segments. There is also excessive generation of reactive oxygen species and induction of oxidative stress that is a major factor that triggers photoreceptor apoptosis [2]. Apoptotic photoreceptor cell death has been examined in feline and rodent models of experimental retinal detachment. Caspase and calpain activation plays an important role in the transduction pathway of the apoptosis cascade, and has been implicated in a number of other ocular diseases [3–6].

Forkhead box O (FoxO) transcription factors are emerging as an important family of proteins that modulate the expression of genes in the regulation of a variety of cellular processes including cell cycle, apoptosis, DNA repair, stress resistance, and metabolism [7]. Activated FoxO proteins promote oxidative stress resistance by binding to promoters of genes encoding manganese superoxide dismutase, catalase, and autophagy-related proteins. These scavenger proteins play an essential role in oxidative detoxification in mammals [8] [9].

Resveratrol is a polyphenolic flavonoid with potent antioxidant activity. Studies show that Resveratrol can play a neuroprotective role in many neurologic disorders such as Parkinson’s disease, Alzheimer’s disease, and hypoxic-ischemic brain damage. This is thought to be through antioxidant and anti-apoptotic mechanisms [10–12]. One recent study found that in a high intraocular pressure induced retinal ischemic injury model, Resveratrol treatment attenuated both ischemic-induced loss of retinal function and reduced ischemia-mediated thinning of the whole retina. This was particularly evident in the inner retinal layers [13].

Our study tested the hypothesis that Resveratrol treatment is neuroprotective to photoreceptor death in experimental RD. We also studied the mechanism by which Resveratrol treatment leads to protection of photoreceptors. This is the first study to investigate the roles of FoxO and Resveratrol in retinal detachment. Moreover, it has the potential to open the door for new therapeutic strategies in many photoreceptor disorders.

## Methods

### Animals

All animal experiments followed the guidelines of the ARVO Statement for the Use of Animals in Ophthalmic and Vision Research and were approved by the Animal Care Committee of the Duke University Institutional Animal Care & Use Committee (IACUC). Adult male Brown Norway rats (weight range, 300–450 g; Charles River Laboratories, Boston, MA) were used in this study.

### Retinal Detachment Induction

RD was created as previously described [14]. The retinal detachment was created in only one eye of each animal (left), with the right eye serving as a control. Briefly, an anterior chamber paracentesis was performed via the corneal limbus to lower intraocular pressure and then approximately one half of the retina was detached by a subretinal injection of 1% sodium hyaluronate (Provisc; Alcon, Fort Worth, TX) into the subretinal space. The other half of the retina remained attached and served as an additional control. Any animals that had surgical complications were excluded from the study.

### Treatment with Resveratrol

The animals were divided into two groups. Resveratrol (3,4′,5-trihydroxy-trans-stilbene, Sigma-Aldrich) was dissolved in 100% ethanol to a concentration of 50 mg/ml. Prior to injection, the Resveratrol/ethanol dose was diluted as follows: 0.05 ml of Resveratrol (50 mg/ml) in 0.2 ml distilled water for a final concentration of 10 mg/ml. Animals were treated starting the day before surgery and daily after surgery with either Resveratrol (20mg/kg) or vehicle control (20% ethanol) via intraperitoneal injection. This dose was chosen because previous studies have shown this to be a safe and effective dosage [15,16].

### TUNEL stain and ONL Thickness Ratio Evaluation

Six rats each group was euthanized 3 days after RD induction and the eyes were enucleated and fixed in 4% paraformaldehyde in phosphate buffer saline (PFA-PBS) at 4^°^C overnight. They were then embedded in Optimal Cutting Temperature media (OCT- Tissue Tek; Sakura Finetec, Torrance, CA), frozen at 20^°^C, and cut in 12mm- thick sections.

A TUNEL assay was performed using DeadEnd™ Fluorometric TUNEL System (Promega, Madison, WI) following a standard manufacturer provided protocol. The number of TUNEL-positive cells was counted in the photoreceptor layer in nine sections per eye. The center of the detached retina was determined and photographed. To be considered TUNEL-positive, each green fluorescent signal had to correspond precisely to the location of a DAPI-stained cell nucleus and had to be significantly brighter than the faint green background of most cell nuclei [17].

The ratio of the ONL thickness to the thickness of neuroretina in the central area of the detached retina was determined by ImageJ software (developed by Wayne Rasband, National Institutes of Health, Bethesda, MD; available at http://rsb.info.nih.gov/ij/index.html) and compared to that in the intraeye control consisting of the attached retina.

### Western Blot Analysis

For Western blot analysis, each group consisted of five animals. The retinas from experimental and control eyes were manually separated from the RPE-choroid at 3 days after inducing retinal detachment. In eyes with retinal detachments, the experimentally detached portion of the retina was identified by direct visualization under dissection microscope and separated from the attached portion of the retina and analyzed separately. Retinal tissue was homogenized in 20mM Tris buffer, pH 7.4 containing 1mM sodium orthovanadate, 0.2 mM EDTA, 0.2 mM PMSF, 0.1 M NaCl, 50 mM NaF, 1× proteaseinhibitor cocktail (Thermo Scientific, Rockford, IL). Protein concentration was determined using Micro BCA Protein Assay Kit (Pierce, Rockford, IL). Equal amounts of protein were separated by Any kD™ or 7.5% Mini-PROTEAN® TGX™ Precast Gels (Bio-Rad Laboratories, CA, USA), and then transferred to a polyvinylidene difluoride (PVDF) membrane (Millipore, Bedford, MA). Membranes were incubated with antibodies against caspase-3, caspase 8 and caspase-9 (1:1000; Cell Signaling Technology, Beverly, MA), FoxO1a, FoxO3a, and FoxO4 (1:1000; Cell Signaling Technology, Beverly, MA), alpha-Spectrin (1:1000; Millipore, Billerica, MA) overnight at 4°C. Bands were detected using the enhanced chemiluminescence reagent (ECL-Plus; Piece, Piscataway, NJ). Membranes were exposed to autoradiographic film (HyperFilm; Amersham), and densitometry was performed. Five eyes were used for each group; except for the alpha-secptrin in which only four eyes were used.

The results are expressed as the mean ± SE. All statistical comparisons were performed with a paired t-test. *P* < 0.05 was considered statistically significant.

## Results

### Calpain activation is involved in apoptotic photoreceptor cell death in detached retinas

**Figure 1:**
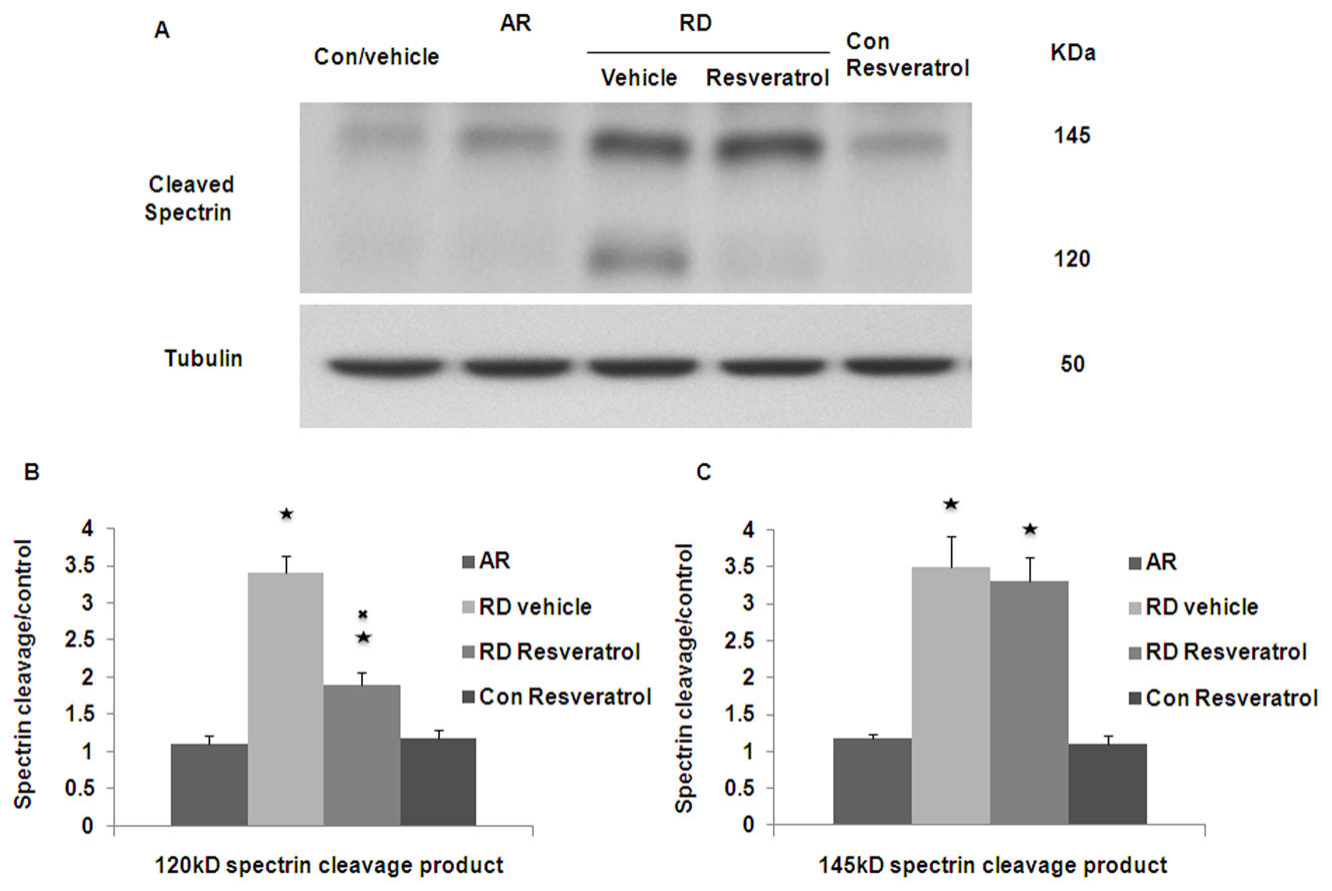
Spectrin cleavage in detached retina. (A) Western blots showing three days after RD, increased expression of 145 and 120-kDa bands in detached retina. Resveratrol treatment only decreases the production of 120-kDa bands. (B) Summary data show a statistically significant increase in the 120-kDa spectrin cleavage product in detached part of retina treated vehicle (RD vehicle) when comparing with attached part of retina (AR) of same eye. Resveratrol treatment significantly decreases 120-kDa spectrin cleavage product (mean ± SEM) in detached retina (RD Resveratrol). (C) Summary data show a statistically significant increase in the 145-kDa spectrin cleavage product in RD vehicle and RD Resveratrol. Resveratrol treatment dose not significantly decrease 145-kDa spectrin cleavage product (mean ± SEM). ★: P < 0.05 statistically significant compare to AR. ✖: P < 0.05 statistically significant compare to RD vehicle.

In order to study whether calpain activation is involved in a rat retinal detachment model, immunoblot analysis was performed using an antibody that recognizes both the caspase specific 120-kDa and the calpain specific 145-kDa fragments of cleaved spectrin. Three days after retinal detachment, there was significantly increased expression of both 145 and 120-kDa bands in all detached portions of the retina. Resveratrol treatment only significantly decreases 120-kDa spectrin cleavage product in detached retina (Figure 1).

### Resveratrol decreases activation of intrinsic and extrinsic caspase apoptotic pathways triggered by RD

**Figure 2:**
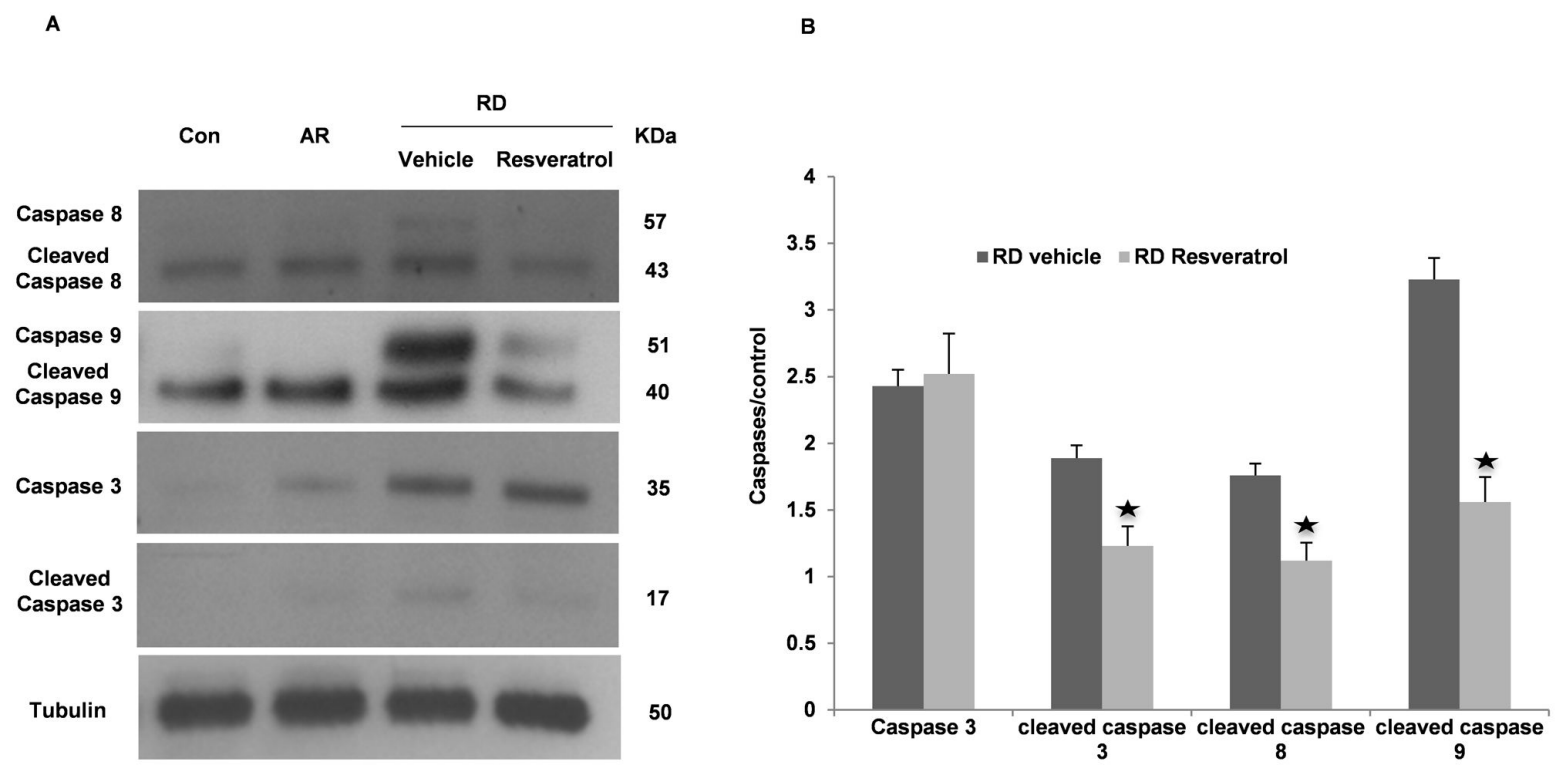
Effect of Resveratrol on levels of Caspase 3, 8 and 9 in RD. (A) Western blots showing three days after RD, increased expression of caspase 3, cleaved caspase 3, caspase 8 and caspase 9 in detached retina. Resveratrol treatment decreases cleaved caspase 3, caspase 8 and caspase 9 production. (B) Summary data show a statistically significant deduction of cleaved caspase 3, caspase 8 and caspase 9 in detached part of retina treated Resveratrol (RD Resveratrol) when comparing with detached retina treated with Vehicle (RD Vehicle) (mean ± SEM). ★: P < 0.05 statistically significant compare to RD vehicle.

We investigated protein level changes of cleaved caspase 8, 9 and 3 in whole retinal lysates from control and experiment eyes 3 days after detachment. The cleaved product of caspase 8 (20 kDa), 9 (35 kDa), and 3 (17 kDa), were only seen in the detached portions of the retina (Figure 2).

To determine the mechanism by which Resveratrol treatment led to protection of photoreceptors, we evaluated the protein level of cleaved caspase 8, 9 and 3 in RD with or without Resveratrol treatment. In rats receiving Resveratrol (20mg/kg), significantly less caspase 8, 9 and 3 were observed 3 days after RD. This represents a significant suppression of both intrinsic and extrinsic pathways (Figure 2). However Resveratrol treatment only decreases the production of the 120-kDa band of spectrin and not the 145-KDa band. This means that Resveratrol treatment decreases *in vivo* caspase activity in RD.

### Resveratrol treatment leads to reduced photoreceptor apoptosis in retinal detachment

**Figure 3:**
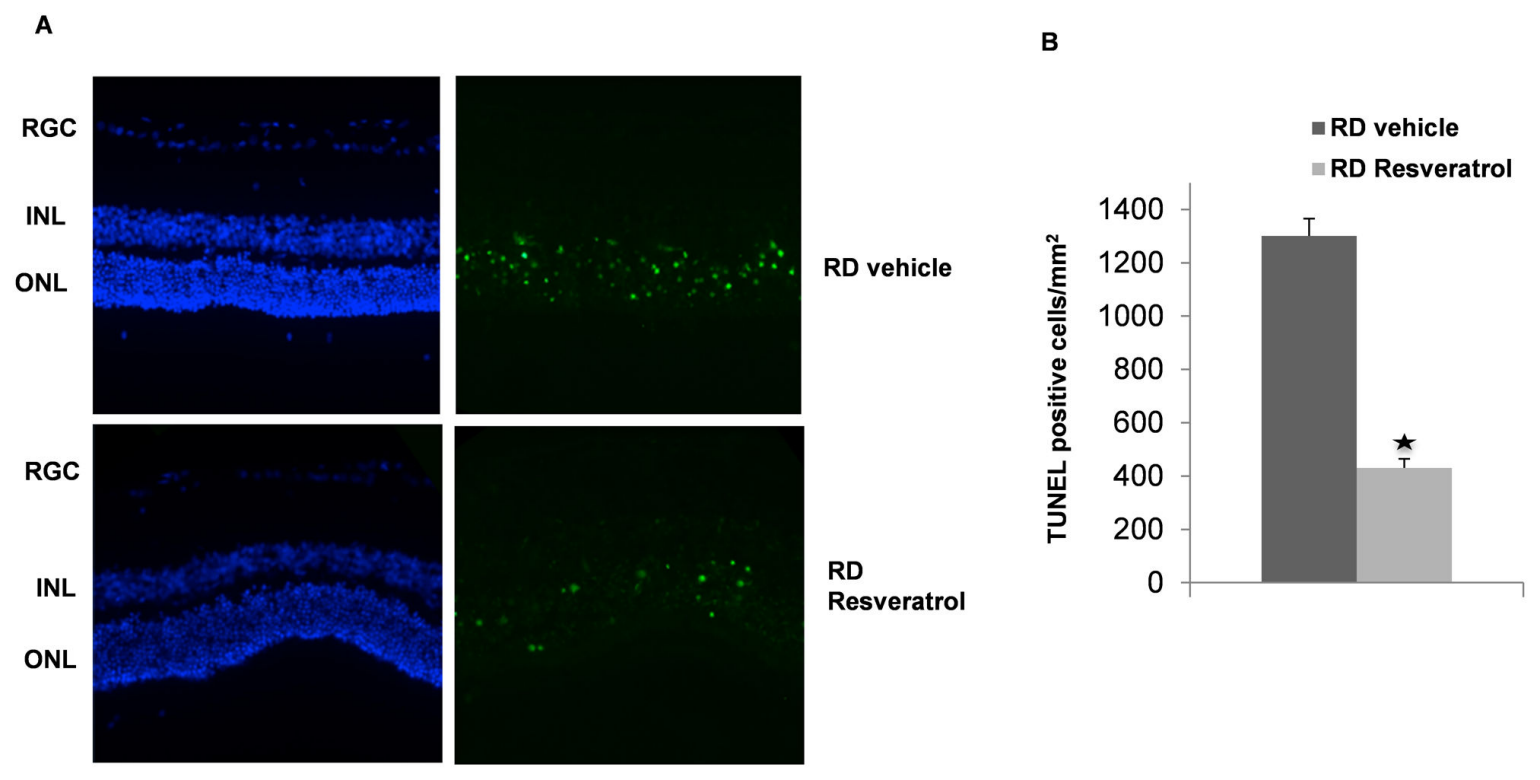
Systemic Resveratrol administration decreases photoreceptor cell loss in RD. (A) DAPI (blue) and TUNEL (green) staining of detached retina sections. (B) Quantitative analysis of TUNEL positive cells 3 days after RD with or without daily treatment (20mg/kg/day, I.P.). Resveratrol treatment significantly decreases TUNEL positive cells (p<0.05, n=6).

We next tested the hypothesis that Resveratrol treatment was neuroprotective to photoreceptor death in experimental RD. First, we assessed photoreceptor death after RD by TUNEL staining which detects DNA fragmentation in apoptotic or necrotic nuclei [18]. Intraperitoneal administration of Resveratrol (20mg/kg) significantly reduced the number of TUNEL-positive cells in the ONL three days after RD from 1301± 51 cells/mm^2^ in the control group to 430± 35 cells/mm^2^ in the treatment group, (P< 0.05, n=6) (Figure 3).

**Figure 4:**
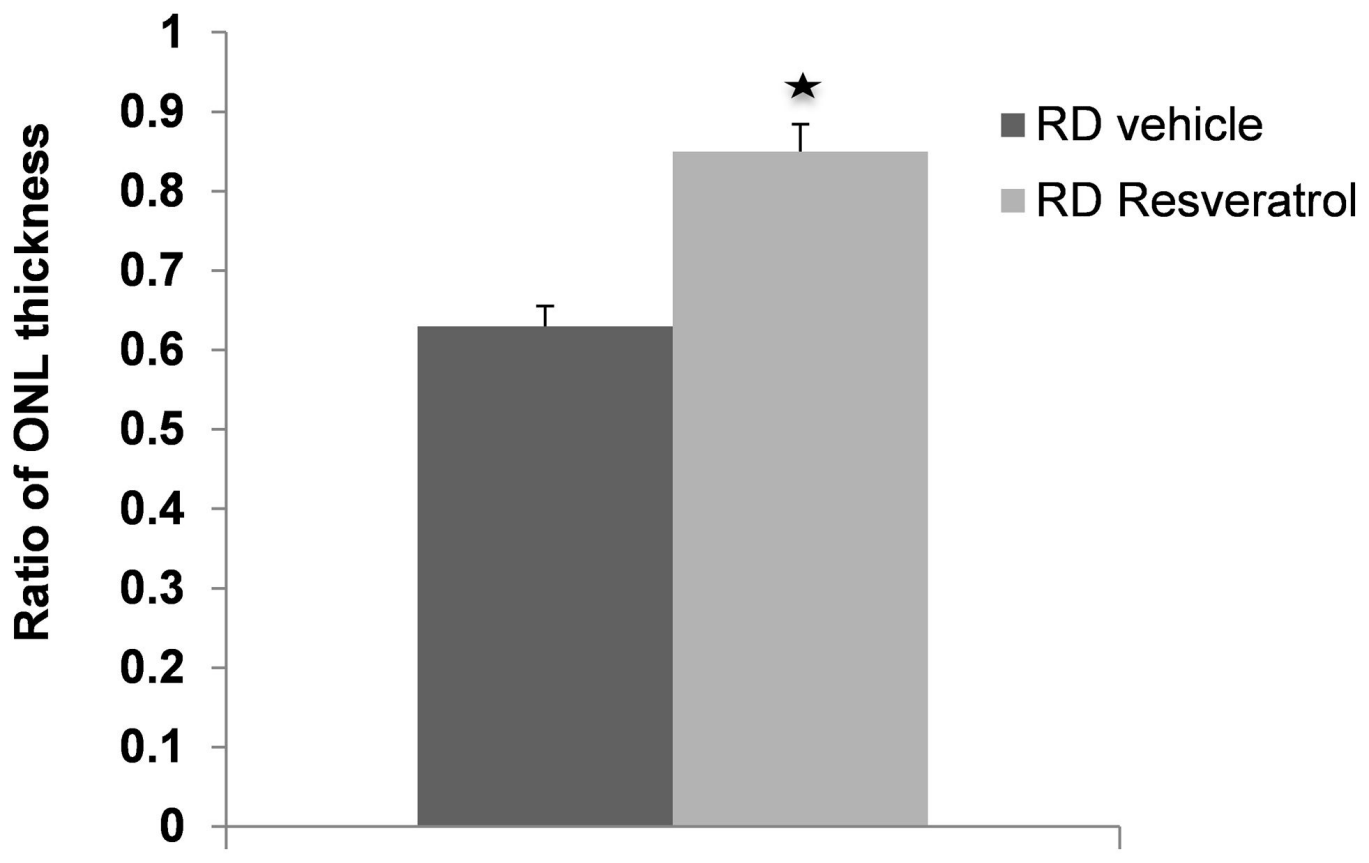
The protective effect of Resveratrol on ONL. Quantitative data exhibiting the protective effect of Resveratrol on ONL thickness preservation 3 days after RD. Resveratrol treatment group has 59% less of ONL thickness loss (p<0.05, n=6).

We then assessed the ability of systemically administered Resveratrol to preserve ONL thickness after retinal detachment. The standardized ratio of ONL to total thickness in detached versus attached areas was measured and compared between vehicle and Resveratrol treated groups. A ratio of 1 represents no loss of ONL thickness, while ratios less than 1 represent loss of ONL thickness. After three days of detachment, the ONL thickness ratio of the vehicle-treated group decreased to 0.63±0.03, while Resveratrol significantly decreased the reduction of ONL thickness ratio (0.85±0.03, P<0.05, n=6). (Figure 4.) Therefore, Resveratrol therapy appeared to be protective against ONL loss in experimental retinal detachment.

### Resveratrol up-regulates the expression of FoxO family protein

**Figure 5:**
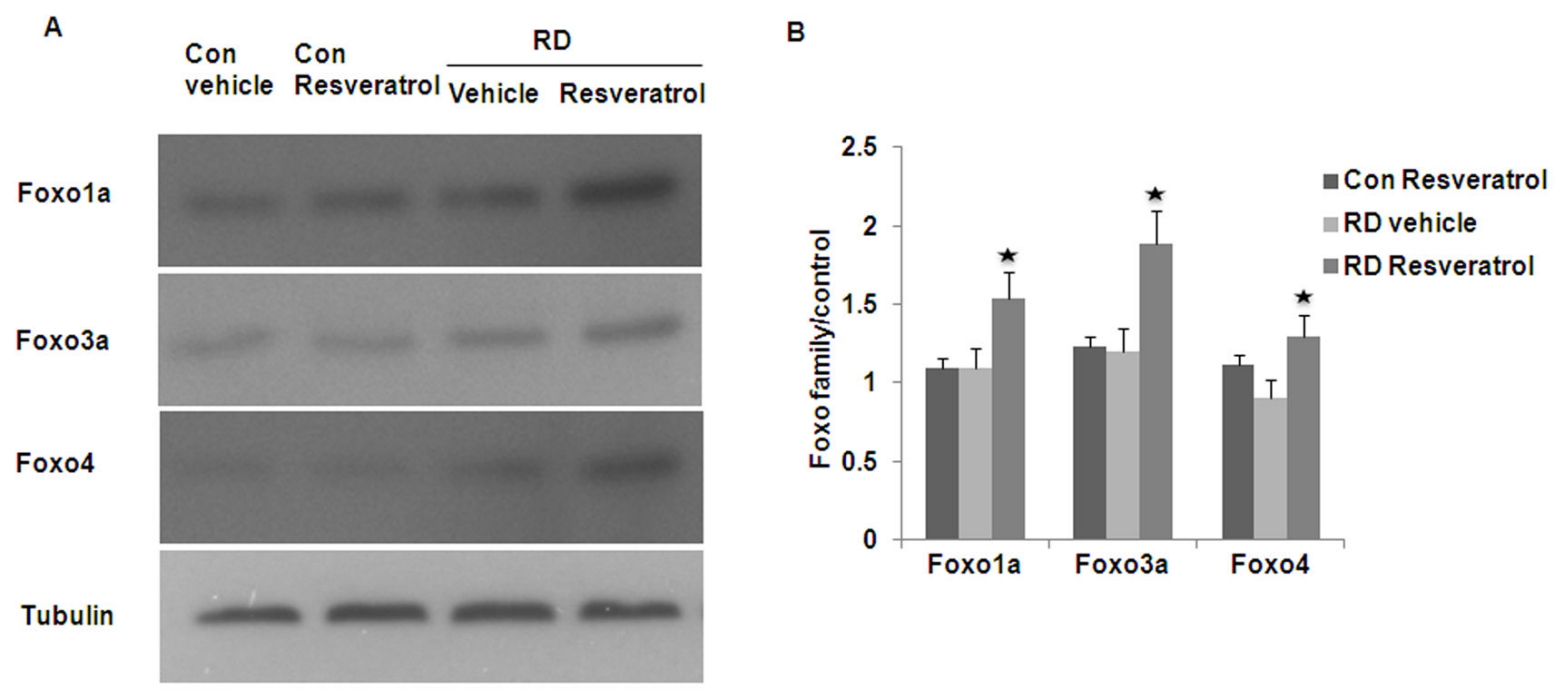
Resveratrol treatment increases FoxO1a, FoxO3a and FoxO4 expression in RD retina. There is baseline expression of FoxO1a, FoxO3a and FoxO4 in attached retina without Resveratrol treatment, Resveratrol treatment doesn’t change FoxO family protein expression (Con Resveratrol). RD alone also does not increase FoxO family protein expression. Summary data show a statistically significant increase of all three FoxO transcription factors protein level in Resveratrol treatment RD retina (RD Resveratrol).

To determine whether Resveratrol prevention of photoreceptor apoptosis in RD was mediated by FoxO activity, we examined FoxO transcription factor protein levels in the attached and detached retina with and without Resveratrol treatment. There were baseline expression of FoxO1a, FoxO3a and FoxO4 in attached retina without Resveratrol treatment. Resveratrol treatment significantly increased the expression of FoxO1, FoxO3a and FoxO4 in the detached retina. RD or Resveratrol treatment alone did not increase the FoxO transcription factors’ protein levels (Figure 5).

## Discussion

Apoptosis has been thought to be the major form of photoreceptor cell death in RD. For the first time we have demonstrated that Resveratrol has protective effects on the apoptosis of photoreceptor cells in an animal model of RD. Previous studies have shown that Resveratrol has anti-apoptotic effects in a variety of diseases [19–21]. The earlier work at our lab showed that in primary human lens epithelial cells culture, Resveratrol increases the expression of FoxO1A, FoxO3A, and FoxO4 under chronic oxidative stress conditions. Resveratrol exerts a protective effect against oxidative damage. When cells transfected with siRNA of FoxO1A, FoxO3A, FoxO4, or the mixtures of all three, led to the loss of Resveratrol anti-apoptotic effects in stressed cells. In this study, we show that Resveratrol dramatically reduces the apoptosis of photoreceptor and decreases the loss of ONL thickness three days after RD in vivo [22].

Oxidative stress has been implicated as a major factor in photoreceptor death in a variety of retinal diseases. The FoxO pathway has emerged as a major convergence point in the cellular response to oxidative stress. We demonstrate that Resveratrol increases the expression of FoxO1A, FoxO3A, and FoxO4 in retinal detachment. This response is consistent with a homeostatic response to protect photoreceptor cells. Baur et al. found that Resveratrol extended lifespan in a high-fat diet mouse model and that it appeared to be dependent on activation of Sir2 [23]. It was further demonstrated that the Sir2 homolog SIRT1 controls the cellular response to stress by regulating the FoxO family [24]. SIRT1 and the FoxO transcription factor FoxO3 form a complex in cells in response to oxidative stress. Furthermore, it has been shown that FoxO-deficient mice (FoxO1, FoxO3A, and FoxO4) exhibit an increase in ROS generation and changes in the expression of ROS-associated genes in hematopoietic stem cell populations [25]. In our study, we found that Resveratrol increased the expression of FoxO3A and also enhanced the expression of FoxO1A and FoxO4 in the detached retinas, suggesting that FoxO1A and FoxO4 may also be involved in the protective effects of Resveratrol. We also found that the Fox, Os expression is not increased in the detached retinas without Resveratrol treatment which indicates that stress alone is not enough to trigger the expression of the FoxO transcription family.

Prior studies report that both intrinsic and extrinsic caspase pathway activation is involved in RD. This subsequently leads to activation of the effector caspases (caspase 3 and 7) and leads to apoptosis of photopreceptor cells [3]. Our results demonstrate that cleavage of both caspase 8 and 9 are observed in RD. Resveratrol treatment blocks the activation of both the intrinsic and extrinsic caspase pathway and this leads to less activation of the effector caspase 3.

Calpains are ubiquitously expressed calcium-dependent cysteine proteases. Alpha-spectrin is one of the lytic substrates of calpain. Recently calpain activation has also been implicated in a number of eye diseases related to photoreceptor apoptosis [26] and calpain inhibitors have been shown to have protective effects. Spectrin is a cytoskeletal calmodulin-binding protein that is cleaved during apoptosis by calpain and caspases to form distinct products [27]. Calpain cleavage of spectrin results in a 145-kDa fragment, and caspase cleavage produces a 120-kDa product [27]. Here we report increases in both the 120-kDa and the 145-kDa spectrin breakdown products, suggesting that both calpain and the caspases pathways are simultaneously activated in experimental RD. We also show that Resveratrol may prevent photoreceptor cell death in stressed cells by upregulating the FoxO transcription family and inhibit the caspase pathway. Furthermore, Resveratrol has no effect on calpain activation. In other words, Resveratrol likely exerts its protective effect through abolishing the activation of caspases. This observation is potentially relevant to understanding why Resveratrol and other previously reported antioxidant molecules [14,17] have only a partial protective effect against apoptosis. One limitation of our study is the relatively small sample size; however, the results appear to be quite convincing that resveratrol has a protective effect against photoreceptor loss in experimental retinal detachment. Further studies will be needed to characterize these pathways and develop novel therapeutic strategies to target the early molecular events in photoreceptor apoptosis.
